# Dietary supplement of mushrooms promotes SCFA production and moderately associates with IgA production: A pilot clinical study

**DOI:** 10.3389/fnut.2022.1078060

**Published:** 2023-01-09

**Authors:** Yuichiro Nishimoto, Junya Kawai, Koichiro Mori, Tenagy Hartanto, Kaori Komatsu, Toru Kudo, Shinji Fukuda

**Affiliations:** ^1^Metagen Inc., Tsuruoka, Japan; ^2^Mushroom Research Laboratory, Hokuto Corporation, Nagano, Japan; ^3^Institute for Advanced Biosciences, Keio University, Tsuruoka, Japan; ^4^Gut Environmental Design Group, Kanagawa Institute of Industrial Science and Technology, Kawasaki, Japan; ^5^Transborder Medical Research Center, University of Tsukuba, Tsukuba, Japan; ^6^Laboratory for Regenerative Microbiology, Juntendo University Graduate School of Medicine, Tokyo, Japan

**Keywords:** gut microbiome, gut metabolome, short-chain fatty acids (SCFAs), immunoglobulin A (IgA), mushrooms

## Abstract

**Background:**

Mushrooms are rich in dietary fiber, and fiber intake has been reported to increase the levels of short-chain fatty acids (SCFAs). It has also been reported that SCFAs promote immunoglobulin A (IgA) production, indicating involvement in systemic immunity.

**Objectives:**

The objective of this study was to evaluate the effects of mushroom consumption on the amount of intestinal IgA. We also aimed to comprehensively evaluate the gut microbiota and intestinal metabolome and to conduct an exploratory analysis of their relationship with IgA.

**Methods:**

Healthy adults (*n* = 80) were enrolled in a parallel group trial. Participants consumed a diet with mushrooms or a placebo diet once daily for 4 weeks. Gut microbiota profiles were assessed by sequencing the bacterial 16S ribosomal RNA-encoding gene. Intestinal metabolome profiles were analyzed using capillary electrophoresis-time of flight mass spectrometry (CE-TOFMS).

**Results:**

Mushroom consumption tended to increase IgA levels at 4 weeks of consumption compared to those in the control group (*p* = 0.0807; Hedges’ *g* = 0.480). The mushroom group had significantly higher levels of intestinal SCFAs, such as butyrate and propionate, than the control group (*p* = 0.001 and 0.020; Hedges’ *g* = 0.824 and 0.474, respectively). Correlation analysis between the changes in the amount of intestinal IgA and the baseline features of the intestinal environment showed that the increasing amount of intestinal IgA was positively correlated with the baseline levels of SCFAs (Spearman’s R = 0.559 and 0.419 for butyrate and propionate, respectively).

**Conclusion:**

Consumption of mushrooms significantly increased the intestinal SCFAs and IgA in some subjects. The increase in intestinal IgA levels was more prominent in subjects with higher SCFA levels at baseline. This finding provides evidence that mushroom alters the intestinal environment, but the intensity of the effect still depends on the baseline intestinal environment. This trial was registered at www.umin.ac.jp as UMIN000043979.

## 1. Introduction

The immune system comprises a complex network of innate and adaptive components in all tissues and plays an important role in host defense against a variety of potentially harmful external factors as well as endogenous perturbations of homeostasis. Of these components, the intestinal immune system is the most sensitive to the gut microbiota, with diverse cells forming a complex immune network that maintains homeostasis. Among the various immune factors, immunoglobulin A (IgA) is an important factor for maintain the homeostasis of the instestinal mucosal surfaces environment. IgA has been known to regulate the composition of the gut microbiota by binding with some pathogenic bacteria and preventing their growth (1), while it also binds to commensal bacteria and is involved in their colonization (2). In addition, IgA production has been reported to be regulated by gut microbiota and their metabolites such as short-chain fatty acids (SCFAs) and D-amino acids (3–5). Previous mouse studies have shown that SCFAs enhance post-immune IgA responses through several mechanisms. SCFAs accelerate B cell metabolism to generate more energy, such as acetyl-CoA, required for IgA synthesis. In addition, SCFAs control gene expression to synthesis molecules which are necessary for plasma B cell differentiation (6). In another study, it has also been suggested that SCFAs may be associated with IgA production *via* SCFA receptors such as G protein-coupled receptor 43 (GPR43) and GPR109a (7).

The gut microbiota is highly variable in individuals and known to be influenced by multiple factors such as age, degree of obesity and host genetics, which demands cautions to interpret the data especially those involving human subjects (8–10). One way to positively control the gut microbiota in daily life is through diet, especially microbiota-accessible carbohydrates (MACs) (11). The most common MAC is dietary fiber, and in Japan, mushrooms are often used in a variety of recipes and are also well known as a typical source of dietary fiber. Beneficial effects of mushrooms include antiobesity (12), immunomodulatory (13), and antitumor effects (14). The antiobesity effect proceeds through a mechanism, involving changes in the gut microbiota and an increase in SCFA levels (12).

In addition, it has been reported that Japanese people eat a variety of mushrooms, such as *Flammulina velutipes* (Enokitake), *Hypsizygus marmoreus* (Bunashimeji), *Lentinula edodes* (Shiitake), *Grifola frondosa* (Maitake), and *Pleurotus eryngii* (Eringi or King oyster mushroom) (12). Mushrooms are a low-calorie and rich source of dietary fiber, especially β-glucans. Mushrooms such as *Hypsizygus marmoreus* have β-1,3 linked glucan, while *Grifola frondosa* and *Pleurotus eryngii* have not only β1,3 link but also branched with β-1,6 glucosides (15–17). Interestingly, it has also been shown that enriching the diversity of consumed dietary fibers leads to modifications of the gut microbiota and metabolic profile (18). This finding suggests that not only fiber content but also fiber diversity may influence the intestinal environment.

According to previous studies, it is possible that the consumption of various types of mushrooms may contribute to host immune system regulation in relation to SCFA levels and IgA production. In addition, it has been reported that the mushroom polysaccharides act directly on immune cells and show anticancer effects (19). However, there have been no reports of human consumption of various types of mushrooms, especially in terms of their effects on the immunity related to mucosal surfaces homeostasis. In this study, a randomized double-blind placebo-controlled trial was conducted to evaluate the effects of mushrooms on the immune system, especially on IgA content, that occur *via* the gut microbiota and metabolome.

## 2. Materials and methods

### 2.1. Clinical trial

In this study, a randomized, double-blind, placebo-controlled, parallel-group study of Japanese participants was conducted for 4 weeks. The protocol for this clinical trial was approved by the clinical trial ethics review committee of Chiyoda Paramedical Care Clinic (protocol code: MTG21C1, approval date: 2021/04/19, publicly registered at UMIN-CTR, trial number: UMIN000043979). Written informed consent was received from all participants. The test food was mushroom tablets, and the control food was starch tablets. We used three types of mushrooms (*Pleurotus eryngii*, *Grifola frondose*, and *Hypsizygus marmoreus*) that are commonly eaten in Japan. These mushrooms were cultured by the Hokuto Corporation in its facilities (Nagano, Japan). Equal amounts of the fresh fruiting bodies of each mushroom were mixed and boiled in equal volumes of water for 1 h. Then, the hot water-treated mushrooms and the boiled water were freeze-dried and powdered to make the mushroom powder. The test food was 390 mg tablets containing the mushroom powder. The composition and nutrient content of the test foods are shown in Supplementary Table 1. The subjects consumed 27 tablets containing mushrooms or placebo tablets per day for 4 weeks. The trial was initiated in April 2021 and completed in June 2021. The primary outcome was the amount of intestinal IgA. The key secondary outcomes were intestinal microbiota composition, intestinal metabolome composition, stool data (frequency, amount, consistency, color, odor, feeling of incomplete evacuation, and abdominal pain) and clinical blood test results. During the trial, fecal samples were collected at baseline, 2 weeks, and 4 weeks of the dietary intervention period. The collected fecal samples were frozen at −20°C until further expert. In addition, blood samples were collected at baseline and 4 weeks of the dietary intervention period. From the blood samples, we measured total protein, albumin, total bilirubin, ALP, LDH, AST, ALT, γ-GTP, CPK, total cholesterol, triglycerides, high-density lipoprotein cholesterol, low-density lipoprotein cholesterol, urea nitrogen, creatinine, uric acid, sodium, potassium, chlorine, calcium, blood glucose, white blood cell, red blood cells, hemoglobin, hematocrit and platelets.

In total, eighty participants were enrolled for this study. The participants fulfilled the following inclusion criteria: (1) at informed consent, male/female subjects aged more than or equal to 20 and less than 65 years; (2) subjects who had a bowel evacuation 3 to 7 times per week; and (3) those who showed understanding of the study procedures and agreed to participate by providing written informed consent prior to the study. Key exclusion criteria were as follows: (1) subjects who had a plan or who had taken medication within a month before the start of trial, which would affect the trial result; (2) subjects who regularly consumed food for specified health uses, food with function claims, supplements and/or health foods more than thrice per week that would affect the trial results; (3) subjects who regularly consumed mushrooms no less than 6 days per week; (4) subjects who had difficulty restricting mushroom intake to less than 2 days per week, as well as less than 50 g a week; (5) subjects who disliked mushrooms; (6) subjects who had undergone an appendectomy; (7) subjects who received surgery that would affect the trial result within half a year before obtaining consent; (8) subjects who had a previous/current medical history of severe cardiac, hepatic, renal or digestive diseases; (9) pregnant, possibly pregnant, or lactating women; (10) subjects who consumed excessive alcohol; (11) subjects with irregular lifestyles and irregular dietary habits; (12) subjects who had food allergies; (13) subjects who were planning to receive a vaccination during the trial; (14) subjects who were enrolled in other clinical tests with some kind of medicine/food, who took part in those within 4 weeks before this trial, or who joined those after giving informed consent to participate in this trial; (15–17) subjects who donated their blood components and/or whole blood, as indicated–all subjects: 200 ml within the last month, males: 400 ml within the last 3 months, and females: 400 ml within the 4 months prior to this trial; (18) males who had 1200 ml in total of their blood collected within the last 12 months, after adding the blood volumes planned to be sampled during this trial; (19) females who had 800 ml in total of their blood collected within the last 12 months, after adding the amounts of blood planned to be sampled during this trial; and (20) others who were determined ineligible by the principal/sub-principal investigator. Randomization in this trial was performed using the blocked stratified randomization method with the subject assignment manager. Eighty subjects who qualified the inclusion criteria were assigned to two groups (groups A and B) by stratification, taking into consideration the age, male–female ratio, defecation frequency and stool amount. Each group of participants were randomly assigned a symbol “A” or “B” representing the mushrooms and control foods, respectively. Subsequently, assignment table was created with the symbols for the foods and the participant identification code. The assignment table was then sealed and kept tightly concealed by the participant assignment manager. The table was disclosed to the analyst, investigator, and test sharing doctor after data fixation. Eighty subjects were selected for the main trial. All 80 subjects completed the trial. All clinical trial data are given in Supplementary Data 1.

### 2.2. DNA extraction and 16S rRNA gene-based microbiota analysis

DNA extraction from fecal samples was performed as previously reported (20). The fecal samples were initially lyophilized and shaken vigorously using a Shake Master (1,500 rpm, 10 min; Biomedical Science Co., Ltd., Tokyo, Japan). Samples were then suspended in DNA extraction buffer containing 400 μl of a 10% (weight/volume) SDS/TE (10 mM Tris-HCl, 1 mM EDTA; pH 8.0) solution. The fecal samples in the buffer were further shaken with 0.1 mm zirconia/silica beads using a Shake Master (1,500 rpm, 5 min). After centrifugation (17,800 × *g*; 5 min; room temperature), bacterial DNA was extracted using an automated DNA extraction machine (GENE PREP STAR PI-480; Kurabo Industries, Ltd., Osaka, Japan) according to the manufacturer’s protocol. The primers 27F-mod (AGRGTTTGATYMTGGCTCAG) and 338R (TGCTGCCTCCCGTAGGAGT) (21) were used to amplify the V1-V2 region of the 16S rRNA gene. The amplified DNA was sequenced using MiSeq (Illumina, San Diego, CA, USA) according to the manufacturer’s protocol.

### 2.3. Measurement of intestinal IgA levels by ELISA

Freeze-dried fecal samples were disrupted by vigorous shaking as stated previously. Ten milligrams (±0.5 mg) of the disrupted samples were mixed with 1 ml of 1 x PBS. The mixtures were homogenized by a micro mixer (TAITEC) at maximum speed for 5 min, followed by centrifugation at 10,000 × *g* for 10 min. Approximately 800 μl of supernatant was filtered through 0.22 μm syringe filters (Merck, SLGVR33RB) and stored in new 1.5 ml tubes. The amount of intestinal IgA from the filtered supernatant was measured by ELISA using a commercially available quantitative ELISA (Human IgA ELISA Kit, Abcam ab196263). The fecal samples were further diluted by Sample Diluent NS included in the ELISA kit, and the IgA concentration was measured according to the manufacturer’s manual.

### 2.4. Metabolite extraction and D,L-amino acid analysis

Chemical analysis of D,L-amino acids was performed at the Shimadzu Techno-Research, Inc. (Kyoto, Japan). Fifty milligrams of freeze-dried fecal samples were homogenized with 450 ml of methanol and mixed with 500 ml of saline, followed by centrifugation at 9,100 × *g* for 5 min at 4°C. The supernatant was filtered and diluted 10 times with 80% methanol. A stable isotope labeling standard solution kit (NeoSMAAT^®^; Sekisui Medical Co., Ltd., Tokyo, Japan) was used as an internal standard. Then, 100 ml of sample diluent, 100 ml of internal standard solution and 100 ml of chloroform were mixed and centrifuged at 9,100 × *g* for 10 min at room temperature. The supernatant was used for derivatization and determination of D,L-amino acid levels using the method previously reported by Harada et al. (22). Briefly, D,L-amino acids were derivatized with (R)-4-nitrophenyl N-[2’-(diethylamino)-6,6’-dimethyl-[1,1’-biphenyl]-2-yl] carbamate hydrochloride. The derivatized amino acids were determined with reversed-phase liquid chromatography-tandem mass spectrometry (LC–MS/MS). The separation was carried out with a Triart Phenyl column (75 mm × 2.1 mm, 1.9 mm; YMC Co., Ltd., Kyoto, Japan) mounted on HPLC equipment (Nexera X2 series; Shimadzu Corp., Kyoto, Japan) using gradient elution of a mobile phase comprising 0.1% formic acid and 10 mM ammonium formate in water and acetonitrile/water (95:5, v/v). The detection of D,L-amino acids was performed using a triple-quadrupole mass spectrometer (LCMS-8060; Shimadzu Corp., Kyoto, Japan). The measures of the obtained amino acids are available in Supplementary Data 2.

### 2.5. Metabolite extraction and CE-TOFMS-based metabolome analysis

Metabolite extraction from fecal samples was performed as previously described (23). Lyophilized feces were extracted with vigorous shaking in 500 μl of methanol and internal standards (20 μM of methionine sulfone and 20 μM of D-camphor-10-sulfonic acid). The samples were disrupted by intense shaking with 0.1 mm zirconia/silica beads (1,500 rpm, 5 min). Following the addition of 200 μl of ultrapure water and 500 μl of chloroform, samples were further centrifuged at 4,600 × *g* for 15 min and 150 μl of the aqueous layer was transferred to a centrifugal filter tube (Ultrafree MC-PLHCC 250/pk for Metabolome Analysis, Human Metabolome Technologies, Yamagata, Japan). The filtrate was centrifuged and dissolved in 50 μl of ultrapure water immediately before capillary electrophoresis coupled with electrospray ionization time-of-flight mass spectrometry (CE-TOFMS) analysis. The peak from the CE-TOFMS was identified and the relative peak area, which is a value based on comparison with internal standards peak area, was calculated. From the relative peak area, the quantitative values of some of the metabolites were calculated by comparison with reference material. A list of the 922 metabolites identified and their analyzed relative peak area are described in the Supplementary Data 3. The obtained metabolome relative area and quantitative values are available in Supplementary Data 4 and 5, respectively.

### 2.6. Bioinformatics and statistical analysis

We used QIIME2 version 2019.10 for the 16S rRNA gene-based microbiota analysis (24). The primer bases were deleted by cutadapt (25) (options: –p-discard-untrimmed). Subsequently, DADA2 was used for quality filtering and generating amplicon sequence variants (ASVs) (options: -p-trunc-len-f 230 -p-trunc-len-r 130) (26). The ASVs were assigned to taxa by applying classifier for Silva SSU Ref Nr 99 (version 132) (command: “qiime feature-classifier classify-sklearn”; options: default) (27). The microbiota data are given in Supplementary Data 6.

In the statistical analysis of main outcome (intestinal IgA amount), the Hedge’s *g* and its 95% confidence interval was calculated in R (Version 3.6.1) using the “effectsize” package (Version 0.4.4.1). In addition, two-way repeated measures analysis of variance (ANOVA) was conducted on the two independent features, groups (placebo and mushroom groups) and timepoints (0 week, 2 weeks, and 4 weeks), by “car” (version 3.1.1) and “lme4” package (version 1.1.28).

Beta-diversity analysis was performed using weighted UniFrac distance and Bray–Curtis distance calculated from the microbiota amplicon sequence variant (ASV) and metabolome quantitative data, respectively (python 3.7.6 and scikit-learn version 0.20.0). In addition, two-way repeated measures permutational ANOVA (PERMANOVA) was couducted on the two independent features, groups (placebo and mushroom groups) and timepoints (0 week, and 4 weeks), by R “vegan” package (version 2.5.7).

In the differential abundance analysis, Wilcoxon rank-sum test and Wilcoxon signed-rank with Benjamini–Hochberg false discovery rate (FDR) correction was used (scipy version 1.5.2 and statsmodels version 0.10.0, respectively) in the pairwise comparison of the clinical data, relative abundance of intestinal microbes and the relative area of metabolites. Bacteria with mean relative abundance below 0.001 and metabolites undetected in 75% of the samples were excluded for comparison. The enrichment analysis in MetaboAnalyst was used for the categorization of metabolites (28). Of these, categories in which more than three metabolites were classified were extracted. In the analysis of associations among intestinal IgA, microbes, and metabolites, correlation analysis and group comparison were performed. In the correlation analysis, Spearman rank correlation coefficient of the difference in intestinal IgA content between 4-week and 0-week with either (1) baseline values; or (2) differential values of each gut microbe and metabolite abundance and blood measured item were calculated within the test food intake group (scipy version 1.5.2). In the group comparison, Wilcoxon rank-sum test was performed between the top 10 subjects and bottom 10 subjects based on the increase in intestinal IgA within the test food group (scipy version 1.5.2).

## 3. Results

### 3.1. Overview of the clinical trial

**FIGURE 1:**
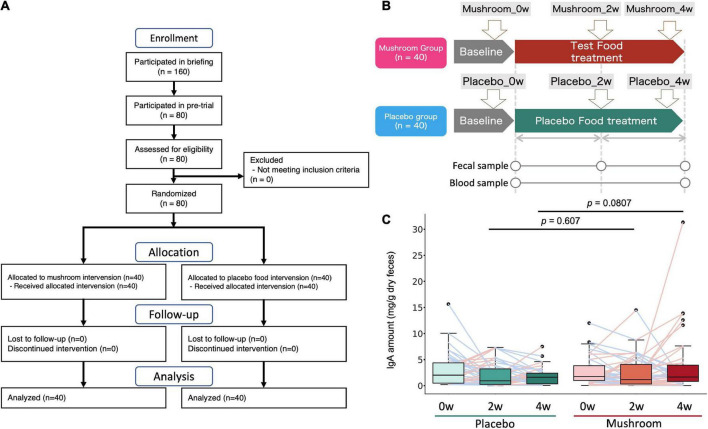
Clinical trial overview and test results for the primary endpoint (intestinal IgA content). **(A)** Flow diagram of the trial. **(B)** Clinical trial diagram and description of time points. Subjects were assigned to either the test food (mushroom) group or the placebo food group, and they consumed the assigned food for 4 weeks. **(C)** Distribution of the primary outcome (intestinal IgA content). The difference between each time point and baseline was calculated and expressed by group and time point. The Wilcoxon rank sum test was performed for comparisons between groups at each intake period (2 or 4 weeks).

To quantify the effects of mushroom consumption, we conducted a randomized, double-blind controlled parallel group trial (Figure 1). Eighty participants were assigned by stratified randomization. Age, sex, frequency of defecation, and stool amount were used as allocation factors. Baseline subject information was analyzed, and the participants’ average age was 41.1 ± 7.1 years. More females were included in the trial than males (67.5% female, 32.5% male). The mean BMI was 21.2 ± 2.6. The mean frequency of defecation was 0.7 ± 0.2 times per day, and the mean stool amount was 2.0 ± 0.9 (counting the number of eggs). Baseline values per group for these statistics were similar in both groups (Table 1). All subjects completed the clinical trial. There was no incidence of side effects. As the primary assessment, influence of placebo/mushroom consumption on intestinal IgA level were evaluated. The two-way ANOVA revealed that there were no significant interaction between the placebo/mushroom intake group, consumption period and IgA level while the consumption period showed the significant difference (*p* < 0.05, two-way ANOVA; Table 2). Although, the pairwise comparison of IgA level at 4-week showed an increasing trend in mushroom intaking group (Table 2, Figure 1C and Supplementary Figure 1; *p* = 0.0807, Wilcoxon rank sum test; Hedges’g = 0.480 (95% CI, 0.04–0.92). Next, a comparison of defecation frequency, a secondary outcome, was conducted. The number of days of defecation tended to increase in the mushroom group (Table 3). The results of other statistical analyses of key secondary outcome data are shown in Supplementary Table 2. Significant differences in creatinine levels were detected between the control and mushroom groups.

**TABLE 1:** Subject information.

	Placebo			Mushroom			*P*-value^*a^
Sex (Men, Women)	13	,	27	13	,	27	−
Age	40.9	±	7.8	41.3	±	6.4	0.815
BMI	21.0	±	2.6	21.5	±	2.7	0.357
Defecation frequency (times/day)	0.8	±	0.2	0.7	±	0.2	0.188
Stool amount^*b^	2.0	±	0.9	2.1	±	1.0	0.877

^*a^*p*-value was calculated by Welch’s *t*-test.

^*b^Stool amounts were determined by counting the number of eggs.

**TABLE 2:** Intestinal immunoglobulin A (IgA) amount.

(mg/g)	Mean and SD^*a^						*P*-value			
	Placebo			Mushroom			Timepoint^*b^	Group^*c^	Timepoint^*c^	Interaction^*c^
0 week	3.1	±	3.2	2.8	±	2.7	−	0.342	0.021	0.364
2 weeks	2.0	±	2.2	2.5	±	3.0	0.607			
4 weeks	1.8	±	1.7	3.8	±	5.9	0.081			

^*a^Mean and Standard Deviation (SD) were expressed by Mean ± SD format.

^*b^Compared between placebo and mushroom groups by Wilcoxon rank-sum test.

^*c^Compared groups (placebo/mushroom), timepoint (0 week, 2 weeks, and 4 weeks), and its interaction by two-way analysis of variance.

**TABLE 3:** Statistical test results for defecation data.

	*P*-value^*a^	*q* value^*b^	Placebo group^*a^		Mushroom group^*a^	
Stool amount^*c^	0.40	0.98	0.09	(0.36)	0.05	(0.37)
Stool consistency^*d^	0.94	0.98	0.23	(0.54)	0.20	(0.51)
Stool color^*e^	0.87	0.98	−0.05	(0.37)	−0.12	(0.58)
Fecal odor^*f^	0.49	0.98	−0.10	(0.34)	−0.06	(0.27)
Feeling of incomplete evacuation^*g^	0.71	0.98	−0.16	(0.41)	−0.09	(0.31)
Abdominal pain^*h^	0.52	0.98	0.04	(0.44)	0.08	(0.35)
Defecation frequency (bowel movement counts/days)	0.30	0.98	0.08	(0.22)	0.12	(0.18)
Defecation frequency (days having a bowel movement/total days)	0.09	0.84	0.68	(2.34)	1.63	(2.23)

^*a^*p*-values were calculated using the Wilcoxon rank sum test. Difference values were calculated for each subject before and after consumption and are shown as the “mean (SD)”.

^*b^*p*-value’s FDR was adjusted by the Benjamini–Hochberg method.

^*c^Stool amounts were determined by counting the number of eggs.

^*d^1. very hard 2. hard 3. somewhat hard 4. normal 5. somewhat soft 6. soft (muddy) 7. very soft (watery).

^*e^1. yellow 2. dark yellow 3. ochre 4. brown 5. dark brown 6. blackish brown.

^*f^1. very weak 2. weak 3. distinct 4. strong 5. very strong.

^*g^1. no residual stool feeling and very refreshing 2. almost no residual stool feeling and somewhat refreshing 3. slightly feeling residual bowel movement; 4. unpleasant with a lingering stool sensation.

^*h^1. a strong pain, 2. a weak pain, 3. almost no pain, 4. no pain at all. SD, standard deviation; FDR, false discovery rate.

### 3.2. Effect of mushroom intake on the gut microbiota and metabolome profiles

**FIGURE 2:**
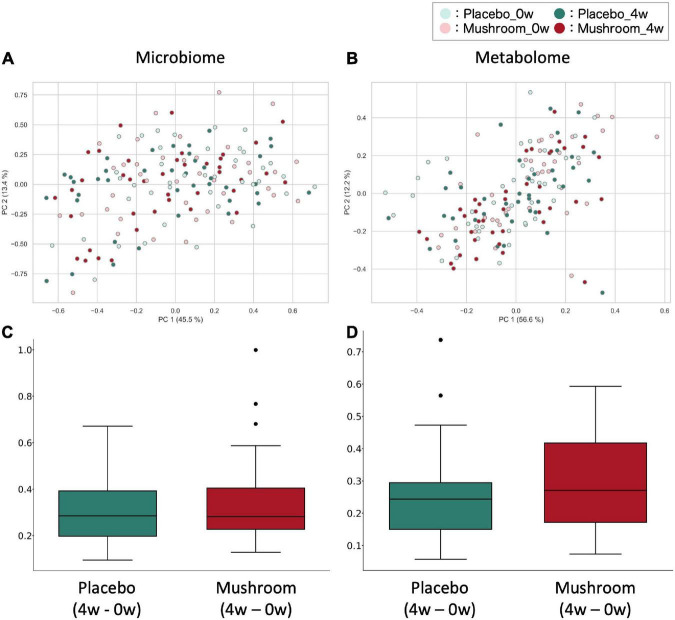
Effect of mushroom consumption on gut microbiota and metabolome profiles. **(A,B)** The **(A)** weighted UniFrac distance for the gut microbiota and **(B)** Bray–Curtis distance for quantitative metabolome profiles among all samples were calculated and visualized by PcoA. **(C,D)** After calculating the **(C)** weighted UniFrac distance of the gut microbiota and **(D)** Bray–Curtis distance of the quantitative metabolome profiles among all samples, the differences in distances before and after ingestion of the same subjects were evaluated and are shown here for each group. No significant differences were detected in either case (Wilcoxon rank sum test).

To quantify the effect of mushroom intake on the gut microbiota and metabolome profiles, 16S rRNA gene-based intestinal microbiota analysis and CE-TOFMS-based intestinal metabolome analysis for fecal samples were performed. Considering the results of the primary outcome, baseline and 4-week fecal samples were analyzed. As a result, a total of 221 bacteria and 478 metabolites were detected. Of these, quantitative values in fecal samples were also measured for 119 metabolites. First, a beta diversity analysis was performed to evaluate the effect on the overall gut microbiota and obtain quantitative metabolome profiles. PERMANOVA analysis showed the significant inter-subjects variations in microbiota and metabolome composition (R2 score is 84.7 and 73.0%, in microbiota weighted UniFrac distance and metabolome Bray–Curtis distance, respectively). Additionally, although the effect size was very small, significant main effect by groups and timepoints were observed on the microbiota composition independently (R2 score, 0.5 and 1.2% for groups and timepoints, respectively). In the metabolome composition, there were no main effects by groups or timepoints observed, however, the interaction effect between the groups and timepoints were found (*p* = 0.008; R2 score = 1.7%) (Supplementary Table 3). The weighted UniFrac distance of the gut microbiota and Bray–Curtis distance of the intestinal metabolome were calculated and compared between the placebo and mushroom groups. There were no significant differences in the microbiota and metabolome distances, which suggested that mushroom consumption had no large effect on the overall microbiota and metabolome profiles (Figure 2).

### 3.3. Effect of mushroom intake on each gut microbe and metabolite

**FIGURE 3:**
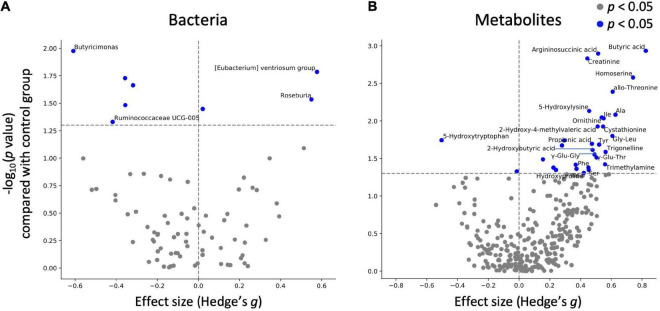
Effect of mushroom intake on gut microbes and metabolites. The *x*-axis represents the effect size (Hedges’ g) of mushroom intake compared with the control group. The *y*-axis represents the logarithm of the Wilcoxon signed-rank test *p*-value compared with the control group. If absolute value of *x*-axis is larger than 0.4 and *p* < 0.05 in *y*-axis, **(A)** bacterium or **(B)** metabolite names were labeled.

Next, statistical tests were performed on each gut microbe and metabolite to evaluate the effects of mushroom tablets. Two control patterns were used in this analysis (after intake for the placebo group and baseline for the mushroom group). As a result, significant differences were detected in the levels of eight bacterial genera and 31 metabolites when compared to those in the placebo group (Figure 3; *p* < 0.05). Common and significant differences were found in two bacterial genera and among 19 metabolites when compared to the pre-consumption and placebo groups (Figure 3 and Supplementary Figure 2). After FDR correction, significant differences were detected for some metabolites only when compared to the baseline of the mushroom group (Supplementary Table 4).

As significant differences were detected for multiple metabolites, categorical enrichment analysis was performed for metabolites for which significant differences were detected in comparison to the baseline. Analysis of the chemical structure category showed that 16 of the metabolites were “amino acids and peptides”, followed by 6 “fatty acids and conjugates including short-chain fatty acids”, 3 “amines”, and 3 “purines” (Table 4).

**TABLE 4:** Enrichment analysis based on chemical categories.

Metabolite set	Total^*a^	Hits^*b^	*P*-value^*c^	Details
Amino acids and peptides	58	16	0.056	Carnosine; Argininosuccinic acid; Cystathionine; Gly; Ala; Ile; Ser; Ornithine; N-Acetylphenylalanine; Leu; Met; Homoserine; Citrulline; 4-Acetamidobutanoic acid; allo-Threonine; γ-Glu-His
Amines	7	3	0.144	Trimethylamine; Spermidine; Triethanolamine
Fatty acids and conjugates	27	6	0.465	2-Hydroxybutyric acid; Propionic acid; Butyric acid; 2-Hydroxy-4-methylvaleric acid; Pelargonic acid; 11-Aminoundecanoic acid
Purines	13	3	0.503	AMP; Xanthine; Paraxanthine

^*a^Number of metabolites used in the statistical analysis for each category.

^*b^Listed those with only hits > 2.

^*c^Compared with other set’s total and hits number.

### 3.4. Increasing D-amino acid levels in the intestine

Mushroom tablet consumption increased the amount of amino acids in the intestinal lumen. Amino acids are converted to the D-form by gut microbes. The D-amino acids, especially D-alanine, contributes to IgA regulation (3). Therefore, we analyzed the amount of D-amino acids, especially in subjects with increased amino acid levels (*n* = 5; 0 and 4 week samples were used). The results showed that L- and D-form amino acids were enriched by mushroom consumption, and the ratio of L-form-to-D-form alanine remained constant (0 w: 1.16 ± 0.15 and 4 w: 1.16 ± 0.09; expressed in the “mean ± SD” format; Supplementary Table 5).

### 3.5. Associations among intestinal IgA, microbes, and metabolites

**FIGURE 4:**
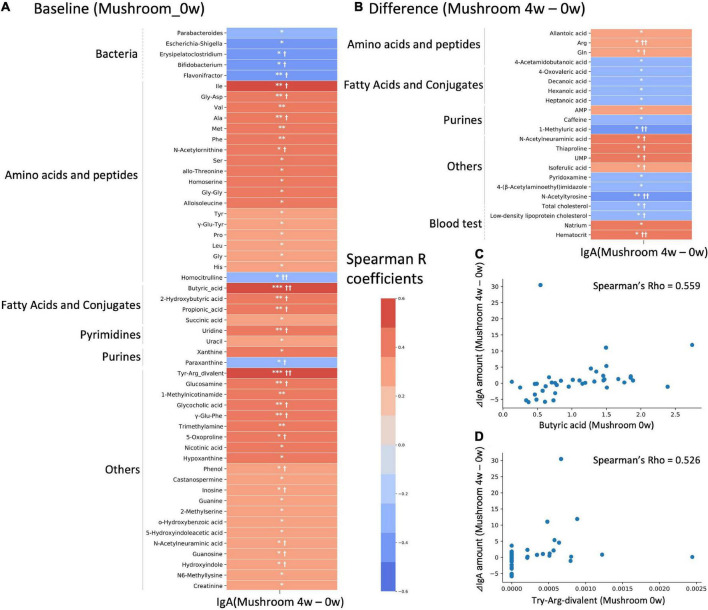
Correlation analysis with intestinal immunoglobulin A (IgA). Heatmap representing the correlation of the difference in intestinal IgA content between 4-week and 0-week with either **(A)** baseline values; or **(B)** differential values of each gut microbe and metabolite abundance and blood measured item. Colors show the Spearman coefficients, and stars show Spearman coefficients’ no-correlation test (*p < 0.05; ^**^p < 0.01; ^***^p < 0.001). In addition, Wilcoxon rank-sum test was performed on the top 10 subjects and bottom 10 subjects based on increase in IgA level (^†^*p* < 0.05; ^††^*p* < 0.01). **(C,D)** Scatter plots showing the relationship of **(C)** butyric acid and **(D)** Try-Arg-divalent to behavior of intestinal IgA level.

There is large inter-individual variation in the degree of response to a diet, often referred to as responder/non-responder, and recent studies have indicated that the gut microbiota composition could be used to identify those subjects who would benefit from dietary interventions (29, 30). In this study, the primary outcome which is the intestinal IgA content, showed an increasing trend following 4 weeks of intake. We hypothesized that the gut microbiota and metabolome might be related to inter-individual variation in the increasing of intestinal IgA. We performed a correlation analysis between the differential values of IgA content (mushroom group, 4–0 weeks) and baseline and differential values of each gut microbe and metabolite abundance and blood measured item in the mushroom intake group (Figures 4A–D). If a significant correlation was found, the factor was determined to be a feature of subjects with increased intestinal IgA levels. As a result, 5 bacterial genera and 48 metabolites were detected as baseline features. No one blood item was detected as baseline features. We found that most identified metabolites belonged to amino acids, peptides, fatty acids and their conjugates, showing positive correlations with IgA (Figure 4A and Supplementary Table 6). In addition, pairwise comparison by Wilcoxon rank-sum test was performed on the top 10 subjects and bottom 10 subjects based on the increase in intestinal IgA within the mushroom group. Results were generally consistent with correlation analysis that the features with significant correlation showed the significant difference in pairwise comparison. We then performed a correlation analysis between the differential values of intestinal IgA content (mushroom group, 4–0 weeks) and the differential values of each gut microbe and metabolite abundance and blood measured item in the mushroom intake group (mushroom group, 4–0 weeks). Although no significant correlation was observed for any of the abundance values of microbes, the abundances of 18 metabolites and four blood item showed significant correlations with differential intestinal IgA levels. Of these, only N-acetylneuraminic acid was found to be a common feature in both analyses (Figure 4B and Supplementary Table 6).

## 4. Discussion

In this study, the effect of the intake of mushrooms on intestinal IgA content was quantified. In addition, comprehensive analysis of the gut microbiota and metabolome profile enabled evaluation of the effects of mushrooms on the gut microbiota and metabolome and an exploratory analysis of their association with intestinal IgA.

No significant differences were detected between the placebo and mushroom groups in the effects exerted on the overall gut microbiota. According to our analysis of individual microbes, there was an increase in that of [*Eubacterium*] *ventriosum* group. [*Eubacterium*] *ventriosum* group is a butyrate-producing bacterium. It has been reported that its relative abundance is reduced in centenarians (31) and increased in obese individuals (32). However, there have been very few studies on this group of bacteria and further studies are needed to examine its effects on the host.

As with the gut microbiota, no significant differences were detected between the placebo and mushroom groups regarding the overall gut metabolome profiles. It would also be difficult for food to have a substantial impact on the overall gut metabolome. However, its analysis on an individual factor scale, revealed significant variation in the levels of many metabolites. Notably, the levels of SCFAs were significantly increased. Butyric acid was particularly increased in this study (Figure 3). Since previous studies have reported that the ratio of propionate level to total SCFAs level were increased by the consumption of oat and barley β-glucans (33). As some of the polysaccharides in mushrooms are known to be naturally bound to proteins (proteoglycans), it is likely that there are differences among the β-glucans by its structure. SCFAs have been reported to have beneficial effects such as improving glucose tolerance, suppressing obesity, inflammation, and cancer progression, and exerting immunostimulant effects through IgA production (4, 34–36). Since the mushrooms used in this study contain β-glucans (*Hypsizygus marmoreus*: β-(1-3)-glucan; *Grifola frondosa*: β-1,6-linked glucan with β-1,3 branches or β-1,3-linked glucan branched with β-1,6 glucosides; *Pleurotus eryngii*: branched 1,3-1,6-β-d-glucan) (15–17), SCFA levels may have increased through the metabolism of β-glucans contained in mushrooms by the gut microbiota. In addition, the results of the categorical analysis showed an increase in some amino acid levels upon consumption of mushrooms (Table 4). Some of the polysaccharides in mushrooms are known to be naturally bound to proteins (proteoglycans). Therefore, it is possible that proteoglycans are degraded by the gut microbiota, followed by protease degradation of proteins by host gastrointestinal enzymes and the gut microbiota. Another notable example is the increasing of trimethylamine (TMA), which is known to be produced by the gut microbiota from choline and carnitine. TMA is metabolized in the liver to trimethylamine N-oxide, which is reportedly associated with atherosclerosis (37). Mushrooms are known to contain choline, indicating that TMA levels may be increased *via* metabolism of mushroom-derived choline (38). However, a previous study has also discussed the potential of SCFA to alleviate hypertension (39). In addition, meta-analysis suggested that mushrooms have favorable effects on lipid profiles and are associated with reduced mean blood pressure (40). Therefore, it is difficult to conclusively associate the increased risk of arteriosclerosis with mushroom intake.

There is large inter-individual variation in the degree of response to diet, and association analysis has identified specific gut microbiota and metabolome features in subjects with increased intestinal IgA levels. In particular, enrichment of SCFAs and some amino acids was characterized in the baseline features. SCFAs have been reported to promote IgA production by activating intestinal B cells (4). However, butyrate is used as an energy source by intestinal mucosal cells, and propionate contributes to gluconeogenesis in the liver (41). As such, their concentration in the colon may need to exceed a certain amount for them to be utilized in IgA production. Regarding amino acids, it has also been reported that D-amino acids produced by the gut microbiota, especially D-alanine, stimulate macrophages and cause IgA production, resulting in increased production of inflammatory cytokines (5). Measurements of L- and D-form amino acids in some samples in this study showed an increase in both L- and D-alanine levels (Supplementary Table 5). Since the L- and D-form ratios of alanine were virtually unchanged before and after ingestion, it is highly likely that D-alanine was also enriched in most subjects. As with the SCFAs, a certain amount of these amino acids is also controlled by the gut microbiota and the host, and it may be necessary to go beyond this control. Considering the results of this study and previous studies, it is possible that intestinal IgA content is increased by increased intestinal D-amino acid levels as well as SCFA levels. In addition, given that SCFA and amino acid levels were increased by mushroom consumption, it is possible that in the long-term this may increase IgA in a larger number of individuals. Validation study of long-term mushroom intake would clarify this possibility.

Next, we quantified the change in the abundances of bacterial genera and metabolites before and after mushroom intake and explored the features of bacteria and metabolites that correlated with the extent of alteration in intestinal IgA content before and after mushroom intake. However, there were few features compared to baseline ones (Figure 4B). N-acetylneuraminic was the only common feature of both analyses. N-Acetylneuraminic acid is the terminal component of glycans in colonic mucins, which is a nutrient for some gut microbes (42), but its relevance to changes in IgA content is unknown.

The following limitations should be considered for our study. First, inspite of our extensive analysis, we could not perform quantitative measurements of bacteria and the large FDR, due to the large number of items measured. Future studies should consider validation tests using murine models to confirm these findings. Second, the analysis is predisposed to more female than male subjects. Because sex-dependent differences in gut microbiota have been reported (43), this demographic characteristics should be taken in further studies, especially in male subjects. Third, single measurement of IgA level may not be sufficient to indicate a healthy gut mucosal environment, thus, other immune parameter such as T-cell dependent high-avidity IgA, which are known to respond to pathogenic bacteria (44), could be the other measurement candidate for further evaluation. T-cell independent IgA may also be the candidate, however, previous study has reported that it has no effect on the composition or diversity of the intestinal microbiota (45). In addition, some species of wild fungi are known to contain heavy metals (46). Although the mushrooms used in this study were cultivated indoors under controlled conditions and tested for heavy metals, we need to be aware of long-term safety of mushrooms.

In conclusion, our work shows that mushrooms have a positive effect on SCFA levels and a partially positive effect on intestinal IgA content. We also found that the subjects that exhibited increased intestinal IgA content were characterized by high baseline SCFA and amino acid levels. Our findings provide an example of stratification of the effects of foods by the intestinal environment.

## Data availability statement

The data presented in this study are deposited in the DDBJ Sequence Read Archive repository, accession number: DRA014974. Clinical, gut metabolome, and gut microbiota raw data are deposited in Supplementary material. The source code used in this study can download at https://github.com/metagen/article_pipeline_MGP012.

## Ethics statement

The studies involving human participants were reviewed and approved by Chiyoda Paramedical Care Clinic. The patients/participants provided their written informed consent to participate in this study.

## Author contributions

JK, KM, TH, KK, and TK: designed research. TH and YN: conducted research. YN: analyzed data or performed statistical analysis. YN, JK, KM, and SF: wrote the manuscript. All authors contributed to the article and approved the submitted version.

## Supplementary material

Supplementary Figure 1 Line graphs of intestinal IgA for each subjects. The line graphs indicate the intestinal IgA amount per timepoint for each subjects.

Supplementary Figure 2 Effect of mushroom intake on gut microbes and metabolites (compared to baseline). The *x*-axis represents the Hedges’ g compared with the baseline. The *y*-axis represents the logarithm of the Wilcoxon signed-rank test *p*-value compared to the mushroom group baseline. If absolute value of *x*-axis is larger than 0.4 and *p* < 0.05 in *y*-axis, **(A)** bacterium or **(B)** metabolite names were labeled.
