# Supplementary figures and images for: Dietary supplement of mushrooms promotes SCFA production and moderately associates with IgA production: A pilot clinical study
Source: Front Nutr. 2023 Jan 9;9:1078060. doi: 10.3389/fnut.2022.1078060 (PMC9868702; doi:10.3389/fnut.2022.1078060)

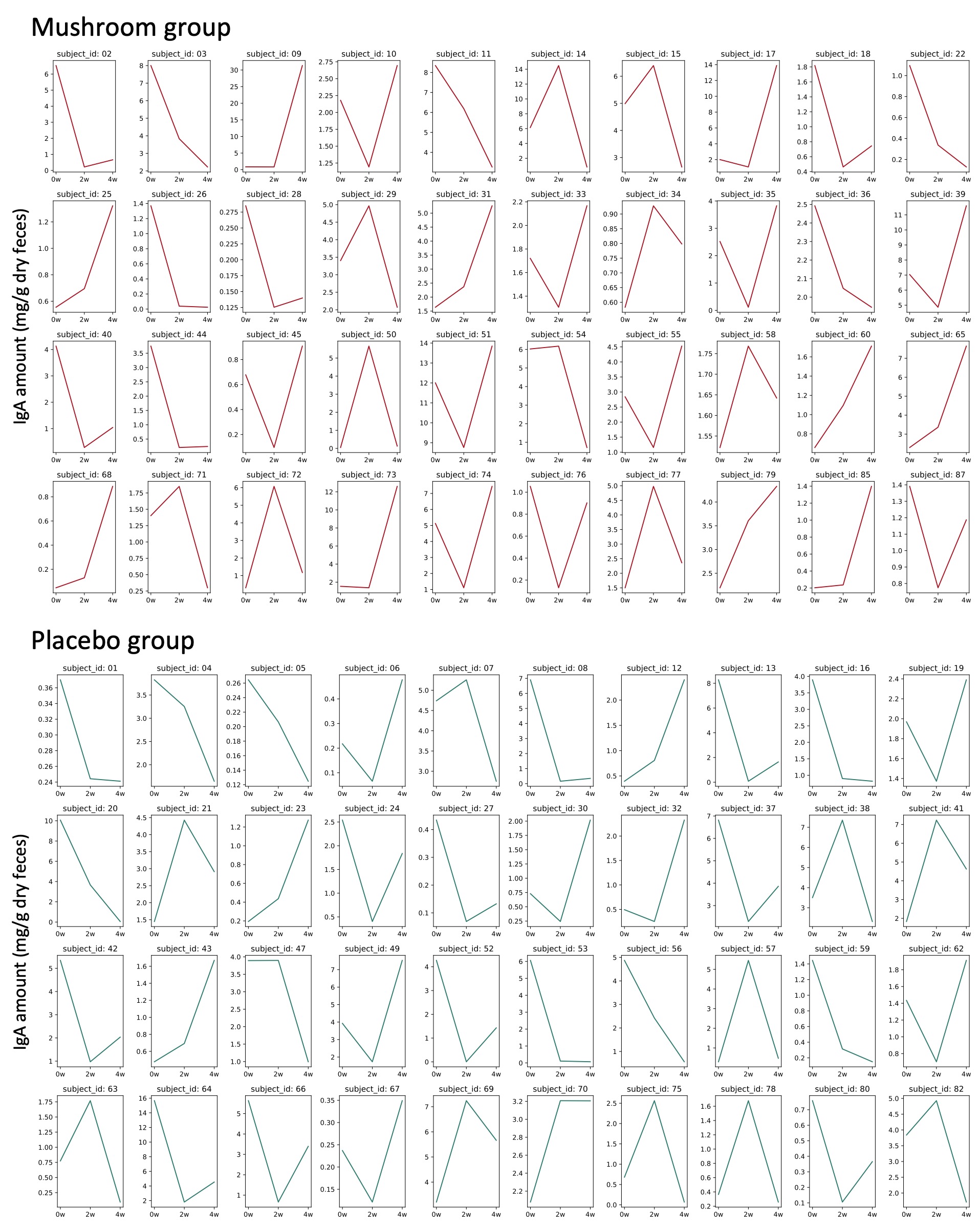

Supplement: Supplementary Figure 1 — Line graphs of intestinal IgA for each subjects. The line graphs indicate the intestinal IgA amount per timepoint for each subjects. [file Image_1.JPEG]

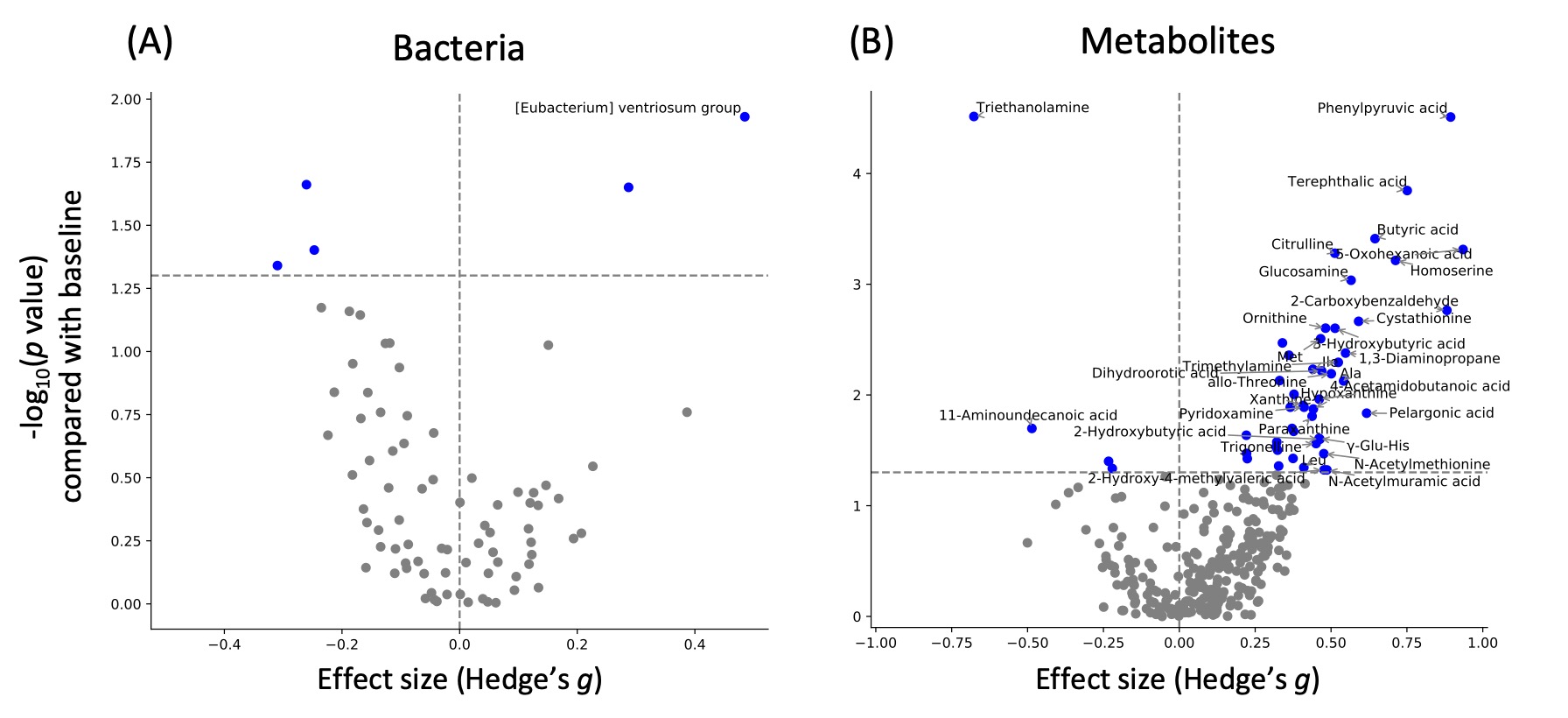

Supplement: Supplementary Figure 2 — Effect of mushroom intake on gut microbes and metabolites (compared to baseline). The x-axis represents the Hedges’ g compared with the baseline. The y-axis represents the logarithm of the Wilcoxon signed-rank test p-value compared to the mushroom group baseline. If absolute value of x-axis is larger than 0.4 and p < 0.05 in y-axis, (A) bacterium or (B) metabolite names were labeled. [file Image_2.JPEG]
